# The Rate of Histamine Degradation by Diamine Oxidase Is Compromised by Other Biogenic Amines

**DOI:** 10.3389/fnut.2022.897028

**Published:** 2022-05-25

**Authors:** Sònia Sánchez-Pérez, Oriol Comas-Basté, Judit Costa-Catala, Irache Iduriaga-Platero, M. Teresa Veciana-Nogués, M. Carmen Vidal-Carou, M. Luz Latorre-Moratalla

**Affiliations:** ^1^Departament de Nutrició, Ciències de l’Alimentació i Gastronomia, Facultat de Farmàcia i Ciències de l’Alimentació, Campus de l’Alimentació de Torribera, Universitat de Barcelona (UB), Santa Coloma de Gramenet, Spain; ^2^Institut de Recerca en Nutrició i Seguretat Alimentària (INSA⋅UB), Universitat de Barcelona (UB), Santa Coloma de Gramenet, Spain; ^3^Xarxa d’Innovació Alimentària (XIA), Barcelona, Spain

**Keywords:** histamine, putrescine, cadaverine, biogenic amines, diamine oxidase (DAO), histamine toxicity, histamine intolerance, histamine degradation

## Abstract

Nowadays, certain uncertainties related to the onset of histamine adverse effects remain unsolved and still require further research. Questions still to be resolved include the wide range of doses at which dietary histamine may trigger symptoms of intoxication (100–10,000 mg/kg) or the appearance of symptoms of histamine intolerance after the consumption of foods presumable without histamine. It seems feasible that other amines, by acting as competitive substrates, could interfere with histamine degradation by the intestinal enzyme diamine oxidase (DAO). Therefore, the aim of this study was to elucidate the interference of different amines on the rate of histamine degradation by DAO. A series of *in vitro* enzymatic assays were performed using histamine as the reaction substrate combined with different proportions of putrescine, cadaverine, tyramine, spermidine, and spermine (1:0.25, 1:1, 1:4, 1:20). Putrescine and cadaverine significantly delayed histamine degradation at all tested concentrations (*p* < 0.001). The greatest effect was observed when putrescine or cadaverine concentrations were 20-fold higher than that of histamine, its degradation being reduced by 70 and 80%, respectively, compared to histamine alone (28.16 ± 1.0 mU). In contrast, tyramine, spermidine and spermine significantly inhibited the histamine degradation rate only at the highest concentration (1:20), reducing it by 32–45%. These results demonstrate that other biogenic amines interfere with histamine metabolization by DAO *in vitro*, the extent depending on the substrate. These findings could explain why susceptibility to dietary histamine is so variable and account for the discrepancies in the scientific databases regarding the amount of histamine that triggers adverse health effects.

## Introduction

According to data from the European Food Safety Authority (EFSA) and the European Center for Disease Prevention and Control (ECDC), histamine is one of the main causal agents of adverse health effects associated with food consumption (1). Histamine intoxication occurs after the ingestion of foods with unusually high amounts of histamine, which overwhelm the intestinal enzymatic capacity for its degradation (2–4). In the last decades, numerous studies have been carried out in this field, but certain uncertainties remain to be clarified, such as why symptoms of intoxication are triggered by extremely variable amounts of histamine in the ingested foods, the reported values ranging from 100 to 10,000 mg/kg (5–11).

The possibility that individuals differ in their histamine-metabolizing capacity has attracted interest in recent years, as it could account for the variable reactions to dietary histamine (2, 3, 12–14). Deficient activity of the intestinal enzyme diamine oxidase (DAO) can explain why some individuals are sensitive to doses of histamine that are safe for the healthy population, the severity of symptoms being correlated with the degree of deficit (12, 13, 15). The inability (or impaired ability) to degrade histamine from food is a clinical disorder known as histamine intolerance. Histamine intolerance, beyond helping to resolve the aforementioned uncertainties, includes a wide range of non-specific gastrointestinal and extra-intestinal symptoms (dermatological, respiratory, neurological, and hemodynamic), due to the ubiquitous distribution of the four histamine receptors in organs and tissues (4, 15, 16). Despite the similarity of certain symptoms, histamine intolerance differs from allergy in that no immune mechanism is involved. DAO deficiency may have a genetic, pathological, or pharmacological etiology. Another explanation, proposed recently, is that it is caused by dysbiosis of the intestinal microbiota, although this hypothesis still needs further study (17–21).

On the other hand, there is evidence that the copresence of other biogenic amines could enhance the adverse effects of histamine (14, 22–24). For example, dose-response studies have found that pure histamine triggers symptoms of intoxication at far higher doses than the histamine ingested from spoiled fish, which may contain other amines, such as putrescine and cadaverine (2, 14, 22). Moreover, according to a recent critical review of different low-histamine diets, certain foods without histamine but containing high levels of other biogenic amines could be responsible of the onset of clinical symptoms in histamine-intolerant individuals (16). Thus, it seems feasible that these other biogenic amines, especially diamines, could compete for deamination by DAO in the intestines, and thus impede histamine degradation, with more noticeable effects in susceptible individuals. However, the available experimental data about the effect of other biogenic amines on histamine degradation are scarce and outdated (25–28). Therefore, the aim of the present study was to elucidate if different amines interfere with the degradation rate of histamine by DAO. For this purpose, a series of *in vitro* enzymatic assays were performed using histamine as the reaction substrate combined with different proportions of putrescine, cadaverine, tyramine, spermidine, and spermine.

## Materials and Methods

### Reagents and Chemicals

Histamine dihydrochloride, putrescine dihydrochloride, cadaverine dihydrochloride, tyramine hydrochloride, spermidine trihydrochloride and spermine tetrahydrochloride, and purified DAO from porcine kidney were purchased from Sigma-Aldrich (St. Louis, MO, United States). UHPLC-grade methanol and acetonitrile, hydrochloric acid 0.1 M, perchloric acid 70%, sodium di-hydrogen phosphate anhydrous, and di-sodium hydrogen phosphate anhydrous were obtained from PanReac Química (Castellar del Vallès, Spain). Acetic acid, boric acid, 1-octanesulfonic acid sodium salt, ammonium formate, phthaldialdehyde (OPA), and brij^®^ L23 solution were acquired from Sigma-Aldrich (St. Louis, MO, United States), and formic acid, sodium acetate anhydrous, potassium hydroxide and 2-mercaptoethanol from Merck (Darmstadt, Germany). A LaboStar System from Evoqua Water Technologies (Warrendale, PA, United States) was used to produce ultrapure water (18.2 MΩcm).

### Experimental Design

The catabolic activity of porcine DAO was tested on six different amino substrates: histamine, putrescine, cadaverine, tyramine, spermidine and spermine, each at a concentration of 25 mg/L. Additionally, several assays were performed to test the degradation rate of a specific concentration of histamine (25 mg/L) by DAO in the presence of different amounts of the other individual amino substrates. Thus, the resulting substrate proportions assayed in this study were the following (histamine:amino substrate): 1:0.25, 1:1, 1:4, and 1:20. Moreover, additional assays considering a mixture of putrescine and cadaverine were also performed at the same four substrate proportions (the proportion refers to the sum of both diamines in equivalent amounts). Three independent experiments were performed in duplicate for each condition.

### *In vitro* Determination of Diamine Oxidase Activity

DAO activity *in vitro* was determined following the procedure previously described by Comas-Basté et al. (29). This method is based on the measurement of the degradation rate of amino substrates during the oxidative deamination reaction catalyzed by DAO. Briefly, purified DAO (0.14 U/mL) was homogenized in 20 mL of 0.05 M phosphate buffer solution (pH 7.2) and placed in a shaker incubator (Ivymen 100-D, JP SELECTA S.A., Abrera, Spain). The addition of a standard solution of the corresponding amino substrate to the working mixture marked the start of the enzymatic reaction. The mixture was kept at 37^°^C under constant stirring (200 rpm). Aliquots of 500 μL were progressively subtracted every 15 min until 180 min of the assay or until the complete disappearance of the substrate. To stop the enzymatic reaction, 15 μL of 2N perchloric acid solution was added to the working mixture and centrifuged at 27,216 × g for 5 min. The supernatant was filtered through a 0.22 μm GHP filter and stored at 4^°^C until UHPLC-FL analysis. The aliquots underwent chromatographic analysis to monitor the degradation rate of the amino substrates and calculate DAO activity, which was expressed as nmol of degraded amine per minute (mU).

### Statistical Analysis

Statistical analysis of data was performed using IBM SPSS Statistics 23.0 software package (IBM Corporation, Armonk, NY, United States). One-way ANOVA followed by a Tukey’s *post hoc* test was applied to investigate the statistical significance of changes in the enzymatic activity in the different studied conditions. Differences with *p* < 0.05 were considered statistically significant.

## Results and Discussion

### Degradation Rate of Different Amino Substrates by Diamine Oxidase

All the assayed amino compounds acted as substrates of the enzyme DAO of porcine origin (Table 1). The highest rate of degradation by DAO was observed in histamine (28.16 ± 1.00 mU), with significant differences compared to the other tested amines (*p* < 0.001). DAO activity was approximately 50% lower on the diamines putrescine and cadaverine than on histamine, whereas on tyramine, spermidine and spermine it was considerably lower than on histamine, putrescine or cadaverine. These results agree with those of Ignesti et al. who demonstrated that porcine kidney DAO had a higher affinity for histamine than other assayed amines; it was 9 and 12-fold lower for putrescine and cadaverine, respectively, and almost 300-fold lower for spermidine (30).

**TABLE 1:** Enzymatic activity (mU) of porcine diamine oxidase (DAO) (mean ± *SD*) on different amino substrates.

Substrates			DAO activity (mU)
Common name	Chemical name	Chemical structure	
Histamine	2-(1H-imidazol-4-yl) ethanamine	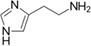	28.16 ± 1.00^a^
Putrescine	Butane-1,4-diamine	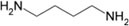	14.11 ± 0.99^b^
Cadaverine	Pentane-1,5-diamine	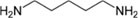	16.20 ± 1.20^b^
Tyramine	4-(2-Aminoethyl) phenol	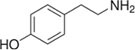	1.77 ± 0.61^c^
Spermidine	N1-(3-Aminopropyl) butane-1,4-diamine	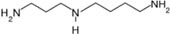	0.65 ± 0.24^c^
Spermine	N1,N4-Bis (3-aminopropyl) butane-1,4-diamine	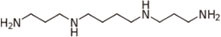	0.58 ± 0.10^c^

*Different letters denote statistically significant differences (p < 0.001).*

The affinity of DAO seems to be influenced by the chemical nature of each compound, especially the molecule length and presence of aliphatic or aromatic moieties (31). According to Blaschko et al. (32), during the enzymatic oxidation of a diamine, the amino substrate is anchored to the enzyme through the two amino groups, forming an intermediate ring compound. It has been suggested that the longer the chain separating the two amino groups, the weaker the bond of the molecule with DAO of animal origin (32).

Substrate preference also seems to depend on the origin of DAO. When extracted from mammals, such as pig, rat or human (30, 33, 34), DAO is reported to have a greater affinity for histamine than for other amines, whereas DAO of plant origin prefers putrescine and cadaverine. Pietrangeli et al. found that DAO from red pea (*Lathyrus cicera* L.) degraded histamine at a slightly lower rate than putrescine and cadaverine (31).

### Interference of Other Amines With Histamine Degradation by Diamine Oxidase

Having demonstrated that other amines frequently found in foods are also substrates of DAO, a quantitative assessment was performed to determine the extent of their interference with DAO degradation of histamine. The competition of these rival substrates for deamination in the intestine would lead to an increase of histamine in plasma, with potentially toxic effects.

**FIGURE 1:**
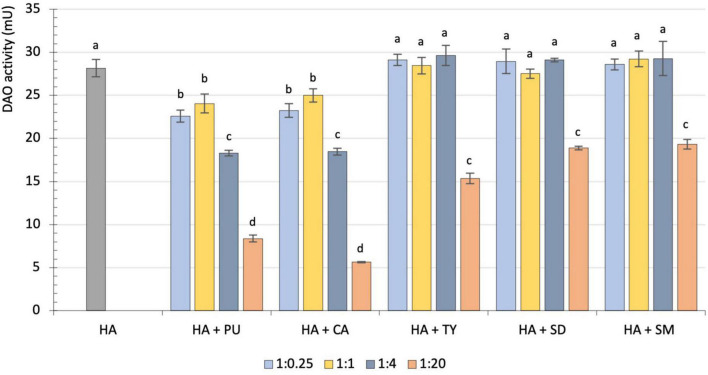
Comparison of diamine oxidase (DAO) activity (mU) on histamine by itself (gray) and on histamine together with different proportions of putrescine, cadaverine, tyramine, spermidine or spermine: 1:0.25 (light blue), 1:1 (yellow), 1:4 (dark blue) and 1:20 (orange). Different letters denote statistically significant differences (*p* < 0.05). HA, Histamine; PU, Putrescine; CA, Cadaverine; TY, Tyramine; SD, Spermidine; SM, Spermine.

The influence of different amounts of putrescine, cadaverine, tyramine, spermidine and spermine on the rate of histamine degradation by porcine kidney DAO is shown in Figure 1. When histamine was assayed together with putrescine or cadaverine, its degradation rate was significantly reduced, regardless of the added amount of these other diamines (*p* < 0.05). When the proportions of putrescine or cadaverine were fourfold higher than histamine, the reduction was approximately 35%, and when 20-fold higher, it increased significantly to 70 and 80%, respectively (*p* < 0.001). Regarding tyramine, spermidine and spermine, no inhibitory effect was observed when these amino substrates were present in concentrations lower than, equal to or fourfold higher than histamine (Figure 1). Only high amounts of tyramine, spermidine or spermine (1:20) significantly inhibited the histamine degradation rate, reducing it by 32–45%. These results confirm that the interference of other biogenic amines with histamine degradation by DAO is directly related to their specific enzymatic activity.

To date, only a few studies carried out in the 1950s and 1980s have assessed the potential influence of competing amino substrates on histamine metabolization by DAO, examining the physiological effects provoked by histamine in animal models (25–28). For example, in 1954 Arunlakshana et al. reported that cadaverine potentiated the contractile effects of histamine in the ileum, trachea and uterus in guinea pigs (26), whereas other compounds added that were not DAO substrates had no impact on histamine metabolization. Lyons et al. reported a 45% increase in histamine absorption in rat intestinal segments when coperfused with cadaverine, accompanied by a 19.6% reduction of imidiazoleacetic acid, the product generated by DAO activity on histamine (28). A few years later, Hui and Taylor similarly reported an increase of unmetabolized histamine in the urinary excretion of rats when putrescine and/or cadaverine were also administered (27), whereas tyramine had no significant effect. Therefore, the current findings confirm these previously reported results, although to the best of our knowledge, the present study is the first to demonstrate the inhibitory effect of other amines (mainly putrescine and cadaverine) on histamine degradation by DAO by directly measuring the enzymatic activity *in vitro*.

**FIGURE 2:**
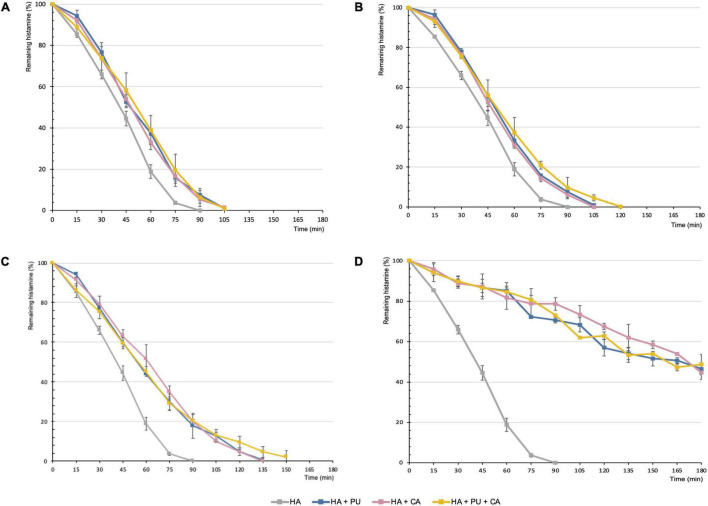
Degradation of histamine (25 mg/L) by itself or with different amounts of putrescine, cadaverine or a mixture of both diamines (sum of equivalent amounts of putrescine and cadaverine). The resulting substrate proportions assayed were the following (histamine:amino substrate): 1:0.25 **(A)**, 1:1 **(B)**, 1:4 **(C)**, and 1:20 **(D)**. HA, Histamine; PU, Putrescine; CA, Cadaverine.

A kinetic study of histamine degradation in the presence of different amounts of putrescine, cadaverine, or a mixture of both diamines (sum of equivalent amounts of putrescine and cadaverine) was performed (Figure 2), as these were the diamines that most compromised the degradation rate of histamine by DAO. Moreover, both substrates are found in food and their formation is frequently linked to the same type of bacteria as histamine.

As can be seen in Figure 2, putrescine and cadaverine had similar inhibitory effects throughout the oxidative deamination of histamine by DAO (*p* > 0.05). Moreover, the concomitant presence of both diamines in the reaction mixture had no significant additional effect (*p* > 0.05). These results can be attributed to both substrates having very similar affinity values for DAO (30).

When histamine by itself (25 mg/L) was exposed to DAO enzyme, it disappeared completely after 90 min (Figure 2). In contrast, in the presence of increasing proportions of putrescine and/or cadaverine, the percentage of histamine remaining at 90 min also increased. Thus, 10% of undegraded histamine was found when assayed with lower or equal amounts of other diamines (Figures 2A,B). This amount increased to 20% when the proportion of putrescine and/or cadaverine was fourfold higher than that of histamine (Figure 2C). Notably, when these diamines were added in the highest proportion (1:20, taking into account the sum of both diamines), histamine degradation was strongly delayed and almost 80% remained at 90 min (Figure 2D). Correspondingly, Mongar reported that the capacity of cadaverine to potentiate the effects of histamine progressively increased with concentration (25). On the other hand, in 1985, Hui and Taylor observed that putrescine or cadaverine had to be administered at levels 4–5-fold higher than histamine to inhibit its degradation by DAO (27), and had no inhibitory effect at an equal dose.

Overall, these results demonstrate that other biogenic amines interfere with histamine degradation by DAO, the extent varying according to the substrate. These findings could help explain the variable susceptibility to dietary histamine and the discrepancies in the literature and scientific databases regarding the amount of histamine responsible for triggering adverse health effects. In fact, certain fermented products (i.e., dry-fermented sausages, cheeses, and plant-fermented products) and semi-preserved fish derivatives frequently contain higher levels of putrescine and/or cadaverine than histamine (16, 35). For example, 71% of samples of dry-fermented sausages and 55% of ripened cheeses were found to have concentrations of putrescine and/or cadaverine at least fourfold higher than histamine. In fermented foods, high amounts of biogenic amines are related to poor hygienic conditions of the raw materials and/or the manufacturing processes, as well as to the aminogenic capacity of the fermentative bacteria (4). In the case of fresh fish, the food category more frequently associated with histamine intoxication outbreaks (In 2020, 100% of these outbreaks occurred at European level were caused by the consumption of fish according to data provided in the joint Zoonoses Report issued by EFSA and ECDC), the occurrence of other biogenic amines is less common than in fermented foods (only 28% of samples had concentrations of putrescine and/or cadaverine at least fourfold higher than histamine) (36). The larger serving size of fish products, together with the extremely high histamine content arising from hygienic defects in their manufacturing or conservation processes, could explain their stronger association with histamine intoxication outbreaks in comparison with fermented products (that often also contain other diamines together with histamine). Furthermore, it is also important to highlight that, in the context of the whole diet, many histamine-free food items are a potential source of significant amounts of other diamines, which could interfere with the intestinal degradation of histamine and enhance its toxicity.

## Data Availability Statement

The original contributions presented in the study are included in the article/supplementary material, further inquiries can be directed to the corresponding author/s.

## Author Contributions

MV-N, MV-C, and ML-M: conceptualization. SS-P, OC-B, JC-C, and II-P: investigation. SS-P, OC-B, and ML-M: writing—original draft preparation. SS-P, OC-B, JC-C, II-P, MV-N, MV-C, and ML-M: writing—review and editing. MV-C and ML-M: supervision. All authors have read and agreed to the published version of the manuscript.

## Conflict of Interest

The authors declare that the research was conducted in the absence of any commercial or financial relationships that could be construed as a potential conflict of interest.

## Publisher’s Note

All claims expressed in this article are solely those of the authors and do not necessarily represent those of their affiliated organizations, or those of the publisher, the editors and the reviewers. Any product that may be evaluated in this article, or claim that may be made by its manufacturer, is not guaranteed or endorsed by the publisher.
